# Quercitrin neutralizes sPLA2IIa activity, reduces the inflammatory IL-6 level in PC3 cell lines, and exhibits anti-tumor activity in the EAC-bearing mice model

**DOI:** 10.3389/fphar.2022.996285

**Published:** 2022-10-17

**Authors:** P. Sophiya, Deepadarshan Urs, Jafar K. Lone, A. S. Giresha, H. Krishna Ram, J. G. Manjunatha, Hamed A. El-Serehy, M. Narayanappa, J. Shankar, Ragini Bhardwaj, Sameer Ahmad Guru, K. K. Dharmappa

**Affiliations:** ^1^ Inflammation Research Laboratory, Department of Studies and Research in Biochemistry, Jnana Kaveri Post Graduate campus, Mangalore University, Kushalanagar, India; ^2^ ICAR-National Bureau of Plant Genetic Resources, New Delhi, India; ^3^ Department of Biochemistry, School of Science, Jain (Deemed-to-be University), Bangalore, India; ^4^ Nisarga Research and Development Trust (T), Bengaluru, India; ^5^ Department of Chemistry, FMKMC College, Mangalore University Constituent College, Madikeri, India; ^6^ Department of Zoology, College of Science, King Saud University, Riyadh, Saudi Arabia; ^7^ Department of Studies in Food Technology, Davanagere University, Davanagere, India; ^8^ Department of Microbiology and Biotechnology, Banasthali Vidyapith, Jaipur, India; ^9^ Department of Development of Biology and Regenerative Medicine, Lurie Children Hospital, Northwestern University, Chicago, IL, United States

**Keywords:** quercitrin, sPLA 2 IIa inhibition, IL-6, ADME-toxicity, inflammation

## Abstract

Human phospholipase A_2_ group IIa (sPLA_2_IIa) is an inflammatory enzyme that plays a significant role in tumorigenesis. Inhibiting the sPLA_2_IIa enzyme with an effective molecule can reduce the inflammatory response and halt cancer progression. The present study evaluates quercitrin, a biflavonoid, for sPLA_2_IIa inhibition and anticancer activity. Quercitrin inhibited sPLA_2_IIa activity to a greater extent—at 86.24% ± 1.41 with an IC_50_ value of 8.77 μM ± 0.9. The nature of sPLA_2_IIa inhibition was evaluated by increasing calcium concentration from 2.5 to 15 µM and substrate from 20 to 120 nM, which did not alter the level of inhibition. Intrinsic fluorescence and far UV-CD studies confirmed the direct interaction of quercitrin with the sPLA_2_IIa enzyme. This significantly reduced the sPLA_2_IIa-induced hemolytic activity and mouse paw edema from 97.32% ± 1.23–16.91% ± 2.03 and 172.87% ± 1.9–118.41% ± 2.53, respectively. As an anticancer activity, quercitrin reduced PC-3 cell viability from 98.66% ± 2.51–18.3% ± 1.52 and significantly decreased the IL-6 level in a dose-dependent manner from 98.35% ± 2.2–37.12% ± 2.4. It increased the mean survival time (MST) of EAC-bearing Swiss albino mice from 30 to 35 days. It obeyed Lipinski’s rule of five, suggesting a druggable property. Thus, all the above experimental results were promising and encouraged further investigation into developing quercitrin as a therapeutic drug for both inflammatory diseases and cancers.

## Graphical Abstract

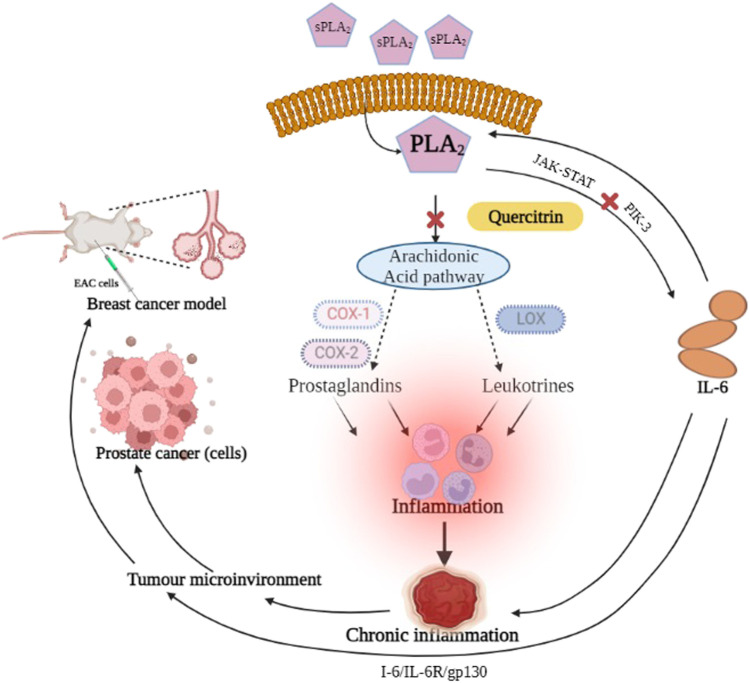

## Introduction

The inflammatory response mediates cell initiation, promotion, malignant conversion, invasion, and metastasis (Mantovani et al., 2008; Aggarwal and Gehlot, 2009; Greten and Grivennikov, 2019). Intrinsic and extrinsic inflammatory pathways mediate cancer progression in risk organs such as the prostate, pancreas, colon, lung, and skin (Mantovani et al., 2008). The environmental effects of cancer and risk factors are associated with chronic inflammation. The known therapy for cancer treatment includes anti-inflammatory drugs like non-steroidal anti-inflammatory drugs (NSAIDs), which imply the significance of inflammation in cancer progression (Waddell and Loughry, 1983; Harris et al., 2005; Rayburn, 2009). Hence, controlling inflammation is an ideal cancer prevention strategy (Todoric, Antonucci, and Karin, 2016).

The sPLA_2_IIa enzyme is a crucial inflammatory enzyme that catalyzes the hydrolysis of membrane phospholipids into arachidonic acid and lysophosphotidate. The arachidonic acid is metabolized into proinflammatory mediators like prostaglandins, prostacyclins, thromboxanes and leukotrienes; the lysophosphotidate is converted into platelet activating factor (PAF) that continues to cause inflammation (Balsinde et al., 2002). The sPLA_2_IIa level has been raised in clinical samples of several diseases, including sepsis, bacterial infections, ARDS (acute respiratory distress syndrome), atherosclerosis, cancers, and trauma (Dore and Boilard, 2019). The proinflammatory prostaglandins and leukotrienes play a significant role in prostate cancer metastasis (Wang and DuBois, 2010) and are overexpressed in human prostate cancers (Dong et al., 2010).

The use of NSAIDs with other drugs for cancer prevention and treatment strategies has demonstrated either decreased cancer incidence or decreased mortality rates (Bardou et al., 2004; Rayburn, 2009). Despite the benefits of NSAIDs, they cause serious side effects like gastrointestinal toxicities, renal injuries, and cardiovascular risk (Hatmi et al., 2006; Bindu et al., 2020). In addition, they cannot regulate the lysophosphatidate metabolism, which continues to cause inflammation (Smith and Langenbach, 2001). Therefore, even though it seems rational, a potent and safe sPLA_2_IIa inhibitor reduces the level of arachidonic acid, lysophosphatidic acid, and their consequent products (Lambeau and Gelb, 2008; Burke and Dennis, 2009).

Potent sPLA_2_IIa inhibitors like varespladib, LY315920, or methyl varespladib were tested in clinical trials but did not demonstrate therapeutic benefits (Nicholls et al., 2012; Nicholls et al., 2014). Drugs like LY315920NA/S-5920, ginkgetin, petrosaspongiolide M, manoalide, and cacospongionolide B were unsuccessful, even though they inhibited sPLA_2_IIa enzyme in nanomolar concentration, which might be due to cytotoxicity or problems associated with formulation. Therefore, the discovery of new potent sPLA_2_IIa inhibitors or the optimization of existing sPLA_2_IIa inhibitors into anti-inflammatory drugs is critical.

There are many benefits to using natural sources as therapeutics over present-day medicines because they have much fewer side effects and are cost-effective (Amin et al., 2021). Traditional medicines are significant sources due to their efficiency against various diseases such as cancer, atherosclerosis, cerebral cardiovascular events, diabetes, hypertension, and Alzheimer’s (Dong et al., 2010; Al-Dabbagh et al., 2018). There are reports that herbal extracts from medicinal plants downregulated the gene expressions of inflammatory markers, including interleukin-IL-1β, tumor necrosis factor-α, transforming growth factor-β 1, nuclear factor kappa-B, and cyclooxygenase-2 (Hamza et al., 2020). Various reports state that plant secondary metabolites and their derivatives have been employed to modulate inflammation and combat cancer (Lin, 2005; Hamza et al., 2020; Harvey, 2000).

In the current study, we have selected *Melastoma malabathricum,* a traditional medicinal plant traditionally used to treat things such as cuts, wounds, hemorrhoids, diarrhea, ulcers, and piles (Lin, 2005). In this study, we concentrated more on secondary metabolites from *M. malabathricum* to validate the traditional usage of this plant to treat various diseases. We initially inspected some pharmaceutically important molecules from *M. malabathricum* by an *in silico* study. Among the inspected molecules, a biflavonoid called quercitrin proved to be a better molecule and was also reported to have several pharmacological properties: anticancer, anti-inflammatory, anti-oxidant (Tang et al., 2019), antibacterial (Cincin et al., 2014), antinociceptive and neuroprotective (Hollman and Katan, 1999), and apoptosis in prostate cancer cell lines (Xu et al., 2013). Therefore, this study tested the efficacy of quercitrin for anti-inflammatory and anticancer activity.

## Materials and methods

### Chemicals and reagents required

Sephadex (G-25, G-50 and G-75), CM—Sephadex C-25, quercitrin, genistein, and lipopolysaccharide (#L2018) were sourced from Sigma-Aldrich. Mouse Anti-Human IL-6 (Cat No: 340527) was sourced from BD Biosciences. Six well cell culture plates were obtained from Biolite - Thermo. The PC3 cell line was obtained from the NCCS, Pune. Diphenyl picryl hydrazyl radical (DPPH), cell culture medium (RPMI 1640, #AL028A), FBS (#RM10432), and D-PBS (#TL1006) were procured from HiMedia Laboratories, Mumbai, India. Ethylenediaminetetraacetic acid (EDTA) and dimethyl sulfoxide (DMSO) were acquired from SRL (Sisco Research Laboratories), India. The solvents and chemicals utilized in the entire investigation were standard laboratory grade.

### Human biological fluid

The Institutional Human Ethical Committee of Mangalore University, India, approved the use of human blood (MU/IHEC/2018/1). Blood samples were collected after obtaining consent letters from healthy volunteers by giving prior information about the study.

### Animals

Swiss albino mice (20–25 g) were used in the neutralization of edema and anti-tumor studies. The animals were handled and supervised according to regulations made under Indian laboratory animal welfare legislation. The approval number of the Institutional Animal Ethical Committee is NGSMIPS/IAEC/NOV-2019/166 (NGSM Institute of Pharmaceutical Science Mangalore).

### Secretory phospholipase A_2_ IIa enzyme

The sPLA_2_IIa was purified according to the method of Kasturi and Gowda (1989), and homogeneity was checked by SDS-PAGE as per Laemmli (1970).

### Molecular docking

The PLA_2_IIa (1POE) structure was retrieved from the Protein Data Bank. The 3D structures of phytoconstituents were taken from the PubChem database. The molecular docking study was conducted using Autodock Vina. The drug molecule was flexible during the docking study, while the sPLA_2_IIa molecule was rigid. The chosen molecule had the lowest binding score with maximum binding affinity. After completing the docking runs, the interactions of different sPLA2IIa residues with inhibitors via hydrogen bonds, hydrophobic interactions, and electrostatic interactions were examined.

### ADME toxicity prediction

The ADME toxicity prediction was conducted using the ADMETSAR server, which included predictions of absorption, metabolism, distribution, excretion, and toxicity (Venkatesh et al., 2017).

### Secretory phospholipase A_2_ assay and inhibition

The sPLA_2_IIa enzyme activity was assessed according to the modified method of Patriarca et al. (1972) by Vishwanath et al. (1993). For the inhibition study, 10 mg of quercitrin was dissolved in 1 ml DMSO and then made up to the required concentration with Tris-HCl buffer. The sPLA_2_IIa inhibition was carried out with 0–18 µM concentrations of quercitrin. Genistein, a known sPLA_2_Iia inhibitor proven as an anti-inflammatory molecule (Dharmappa et al., 2010), was used as a positive control.

### Effect of calcium and substrate concentration on sPLA_2_IIa inhibition

The nature of sPLA_2_IIa inhibition was tested by increasing the calcium concentration from 2.5 to 15 µM and substrate from 20 to 120 nM in the presence and absence of an IC_50_ concentration of quercitrin (separate calcium and substrate-dependent assays were performed).

### Intrinsic fluorescence interaction study

The fluorescence intensity of the sPLA_2_IIa enzyme alone or with quercitrin was measured using the Horiba Jobin Yvon Fluorolog-3 spectrofluorometer. 2 ml of the reaction mixture in a 1 ml path length cuvette consisting of sPLA_2_IIa (20 μg/ml) with different concentrations of quercitrin (0.02–0.1 µM) used in the study. Fluorescence spectra were recorded between 300 and 380 nm and corrected empirically using the tryptophan standard (Prigent-Dachary, 1980).

### Circular dichroism studies

The UV-CD spectra of sPLA_2_IIa (30 μg/ml) was recorded with and without an IC_50_ concentration of quercitrin using a Jasco J-810 spectropolarimeter between 200 and 240 nm. The response time was 2 sec and the bandwidth was 1 nm. A total of 10 scans were made into the final spectrum. The spectrum of the standard reaction mixture was subtracted to correct the spectra.

### Neutralization of indirect hemolytic activity

The assay was carried out according to the method of Boman and Kaletta (1957); 1 ml of RBC and 1 ml of egg yolk in 8 ml of PBS were mixed and used as substrate. The quercitrin was preincubated with sPLA_2_IIa enzyme at 37°C for 30 min and then 1 ml of the substrate was added and incubated at 37°C for 45 min. We then added 9 ml of ice-cold PBS to halt the reaction, which was then centrifuged at 1,500xg for 20 min. The hemolytic activity of the sPLA_2_IIa enzyme in terms of released hemoglobin was measured at 530 nm.

### Neutralization of edema-inducing activity

The method of Yamakawa et al.(1976) altered by Vishwanath et al.(1987) was followed. The sPLA_2_IIa (5 µg) alone or with quercitrin (ranging from 0 to 18 µM) in a total volume of 20 µL was injected into the right hind footpad of the mice; 20 µL saline was injected into the respective left hind footpad as normal control. The animals were euthanized after 45 min by anesthesia (30 mg/kg of pentobarbital i.p.). The limbs were amputated at the ankle joint and were weighed individually.

Edema Ratio=weight of the edematous leg/weight of normal leg (saline injected)×100.

### MTT (3-(4, 5-dimethylthiazol-2-yl)-2, 5-diphenyltetrazolium bromide) assay

The protocol of Alley et al. (1988) and the MTT cell proliferation instruction guidelines were used to determine prostate cancer (PC3) cell viability. The cells were grown in T-25 flasks, harvested, and plated in a 96-well plate in DMEM at a cell density of 10,000 cells per well. After 24 h incubation, the cells were treated with different concentrations of quercitrin. After 24 h incubation, the MTT reagent was added to a 0.5 mg/ml concentration and incubated in the CO_2_ incubator for 3 h. The media containing excess MTT was removed and formazan crystals were dissolved using 100 µL DMSO. A reading was taken at 570 nm for the purple color formazan solution and at 630 nm to subtract the background reading. The percentage viability of PC3 cells was calculated.

### Antitumor activity

The anti-tumor activity of the quercitrin was evaluated using the Ehrlich ascites carcinoma (EAC)-bearing mice model (Raihan et al., 2012). The quercitrin was tested for anti-tumor activity. In brief, 24 mice of either sex were grouped into four sets containing six animals each. Except for Group 1 (normal), they were injected with (*i.p.*) 1×10^6^ EAC cells. Group 1 received only buffers and served as vehicle control, and Group 2 served as disease control; Group 3 animals received quercitrin (10 mg/kg, *p.o.*) and Group 4 served as the positive control. Animals were treated after 24 h of tumor induction and continued for the next 10 days. Experimental animals were observed for clinical signs and mortality. The weight of the animals was recorded and the percentage increase in mean survival time (% MST) was calculated.

% MST=1−TC X100.

where C = mean survival time of the control group; T = mean survival time of the treated group.

### Estimation of interleukin 6 by flow cytometry

The IL-6 level was measured by following the manufacturer’s instructions (BD Biosciences, 2002) for PE Mouse Anti-Human IL-6 (Cat No: 340527) and analyzed by FACS—CellQuest Pro software.

### Statistical analysis

The obtained test results were given as the mean standard deviation (*n* = 3). The IC_50_ value was calculated by Graph Pad Prism version 9.0 (La Jolla, USA) and using one-way ANOVA (analysis of variance), followed by Tukey’s test as a single *post hoc* test to compare the mean obtained from the experiment. The results are all shown with a mean standard error. If the *p* value is < 0.05, then statistical significance is assumed.

## Results and discussion

**FIGURE 1:**
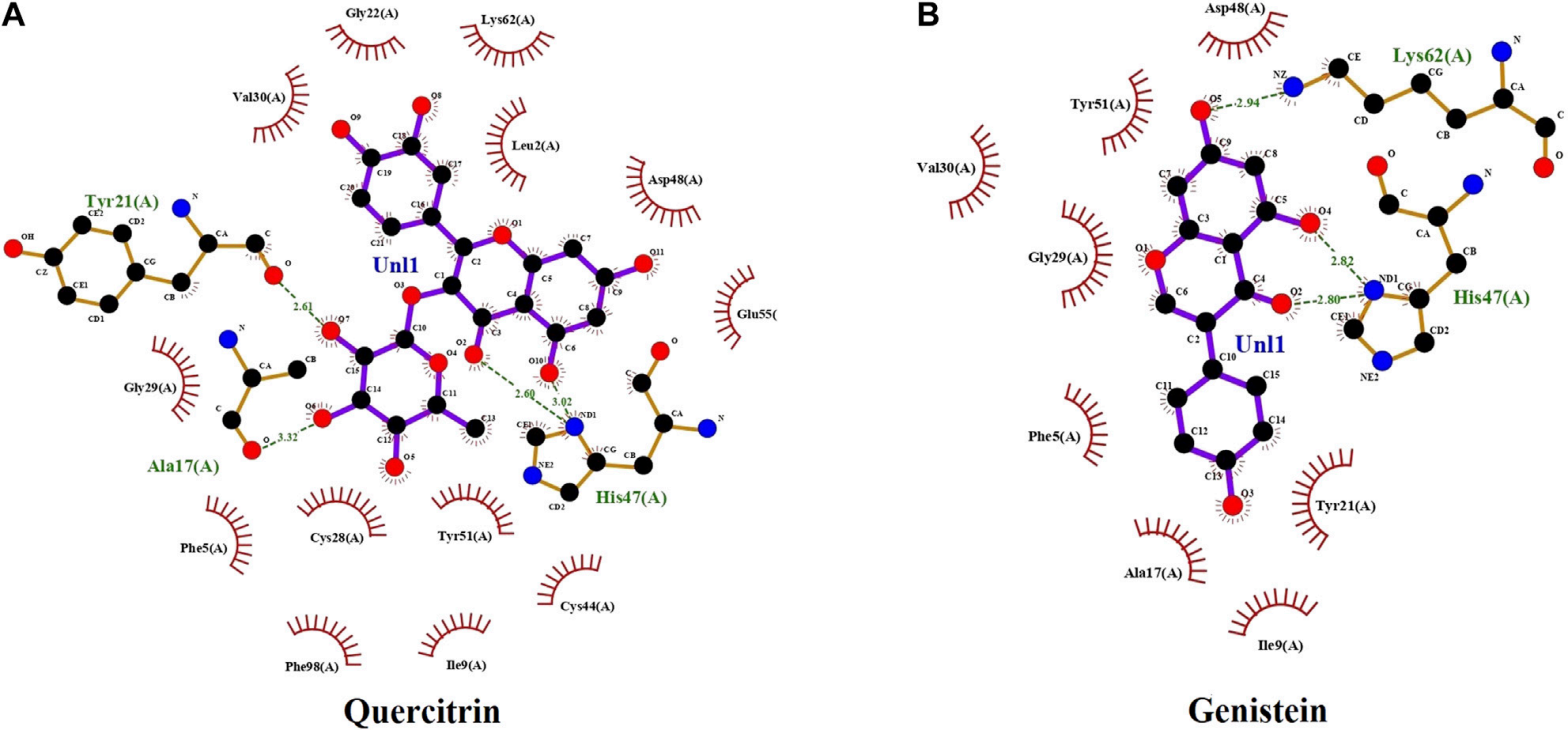
The docking study: docked images of quercitrin **(A)** and genistein **(B)** with sPLA_2_IIa enzyme (1POE).

The computational advances significantly influenced the drug development process because of time saving and cost-effectiveness (Lin et al., 2020). Hence, some pharmaceutically important molecules from the *M. malabathricum* plant—hexadecanoic acid, phthalic acid, desulphosinigrin, quercitrin, and brevifolincarboxylic acid—were subjected to computational study (docking study). AutoDock4 (v4.2.6) was used to analyze the binding pattern and energy values of molecules with human sPLA_2_IIa (1POE). Among the above molecules, quercitrin showed a greater energy value of −8.31 than standard genistein (−7.96) (Table 1). Quercitrin (PubChem ID 5280459) interacts with Ala17, Tyr21, and His47 residues of the sPLA_2_IIa enzyme (H bond). Similarly, genistein (PubChem ID 5280961) interacts with His47 and Lys62 residues of the sPLA_2_IIa enzyme (Figure 1). Any ligand that binds to His 47, Asp48, and/or Ca^2+^ binding loop limits the sPLA_2_IIa activity because they are most important for its catalysis.

**TABLE 1:** Molecular docking of selective bioactive molecules and their energy values against sPLA_2_ IIa (1POE) by Auto Dock Tools 4.2. HBA: hydrogen bond acceptor; HBD: hydrogen bond donor; logP: lipophilicity; MR: molar refractivity; MW: molecular weight in daltons; TPSA: topological polar surface area in Å.

Compound ID	Compound name	HBA	HBD	logP	MR	MW	TPSA	H Bond	No of H bonds	Energy
520159	Hexadecanoicacid, methyl ester	5	3	2.5547	88.6034	318.448	86.99	Gly31	1	−3.66
5280459	Quercitrin	11	6	0.9269	107.1049	447.369	193.11	Ala17, Tyr21, His47	2	−**8.31**
5280961	Genistein	5	3	2.5768	73.989	270.236	90.9	His47, Lys62	1	−**7.96**
6423815	Pthalic acid	4	0	5.0167	97.071	334.449	52.6	Gly29, His47	2	−7.68
9601716	Desulphosinigrin	8	5	1.1167	65.3337	279.310	148.04	Gly29, His47	2	−6
9838995	Brevifolincarboxylic acid	8	4	0.6644	68.1783	292.197	145.27	His47, Gly29, Asp48	3	−7.38

The *in silico* ADMET study was carried out to check the pharmacokinetics and pharmacodynamics properties of the inhibitors. The ligand physical descriptors and pharmaceutically essential properties were analyzed using the ADMETSAR server. The parameters used in ADME-Toxicity prediction are based on Lipinski’s rule of five; accordingly, the quercitrin showed 51.083% of human intestinal absorption −1.277 (log BB) of blood-brain barrier (BBB) permeability, whereas standard genistein showed 93.387% of human intestinal absorption −0.71 (log BB) of the blood-brain barrier. Both compounds did not show AMES toxicity. Thus, the compound quercitrin displayed drug-like qualities.

**FIGURE 2:**
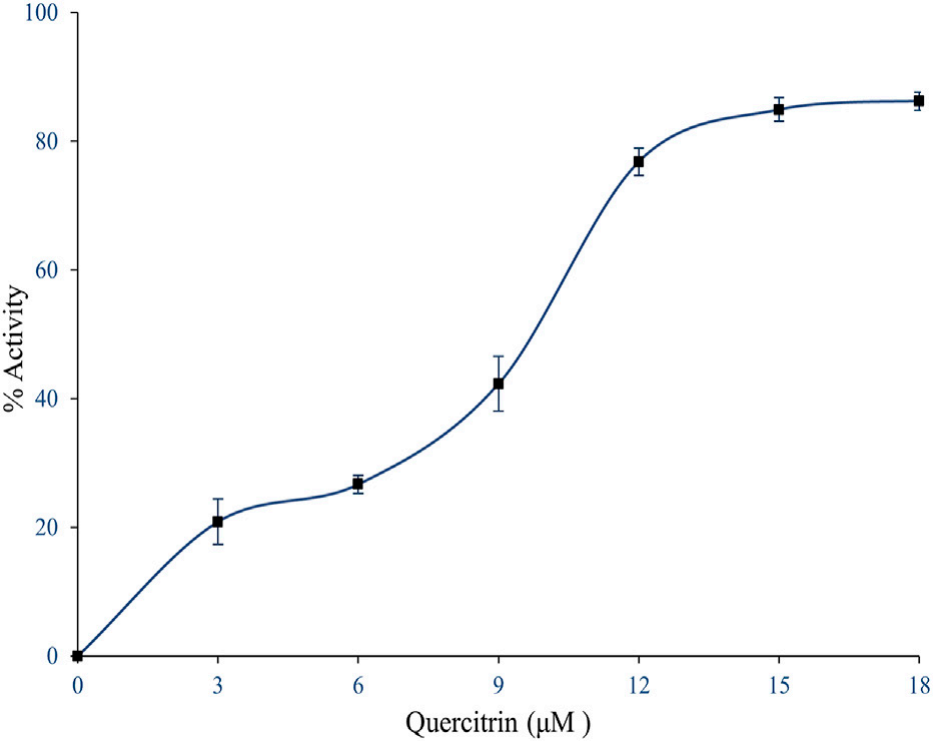
Inhibition of sPLA_2_IIa enzyme by quercitrin. In brief, 350 µl reaction mixture consists of 3.18×10^9^ autoclaved *E. coli* cells, 5 mM calcium, and 100 mM Tris–HCl buffer pH 7.4 with sPLA_2_IIa enzyme and indicated concentration of inhibitors, incubated at 37°C for 60 min. The sPLA_2_IIa activity was estimated by measuring ^14^C radiation using the Quantulus 1220 liquid scintillation spectrometer. sPLA_2_IIa inhibition was noted as a percentage of control and the IC_50_ value was found to be 8.77 μM ± 0.9. The data showed mean ± SD (*n* = 3).

The quercitrin showed better binding energy (negative E value) and exhibited drug-like qualities. Hence, quercitrin was further employed for sPLA_2_IIa inhibition *in vitro*; it better inhibited the sPLA_2_IIa enzyme in a concentration-dependent manner (Figure 2). It inhibited sPLA_2_IIa activity to 86.24% ± 1.41 at 18 µM concentration with an F-statistic value of 0.018 and *p* value of 0.9981. The IC_50_ value of quercitrin was calculated to be 8.77 μM ± 0.9, whereas standard genistein is 5.75 μM ± 1.16. The IC_50_ value of sPLA_2_IIa inhibition was carried out with GraphPad Prism software 9.0.

**FIGURE 3:**
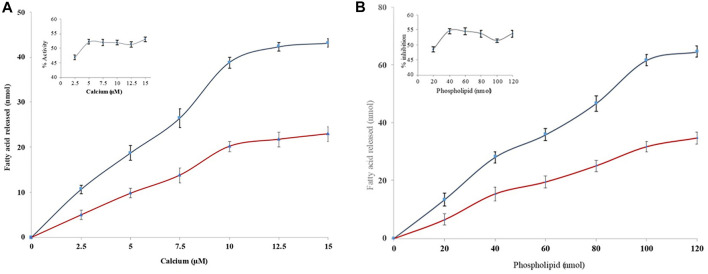
Effect of calcium and substrate concentration on sPLA_2_IIa inhibition. **(A)** sPLA_2_IIa activity without quercitrin (blue line) and with quercitrin (red line) with the indicated concentrations of calcium (2.5–15 µM). **(B)** sPLA_2_IIa activity without quercitrin (blue line) and with quercitrin (red line) by increasing the substrate concentration from 20 to 120 nM. The data are expressed as mean ± standard deviation (*n* = 3).

Many inhibitors halt the sPLA_2_IIa catalysis either by binding the substrate or chelating calcium (Bastian et al., 1993). Hence, the nature of sPLA_2_IIa inhibition was tested by increasing the calcium concentration from 2.5 to 15 µM in the presence and absence of quercitrin (IC_50_ concentration); it showed a constant sPLA_2_IIa inhibition of 48.79% ± 0.8 over all the ranges of calcium (Figure 3A). The sPLA_2_IIa inhibition was also examined by increasing the substrate concentration from 20 to 120 nM in the presence and absence of quercitrin (IC_50_ concentration); it showed a constant inhibition of 47.27% ± 1.0 over all the ranges of substrate concentrations (Figure 3B). The results suggested that sPLA_2_IIa inhibition was independent and did not depend on calcium or substrate concentrations.

**FIGURE 4:**
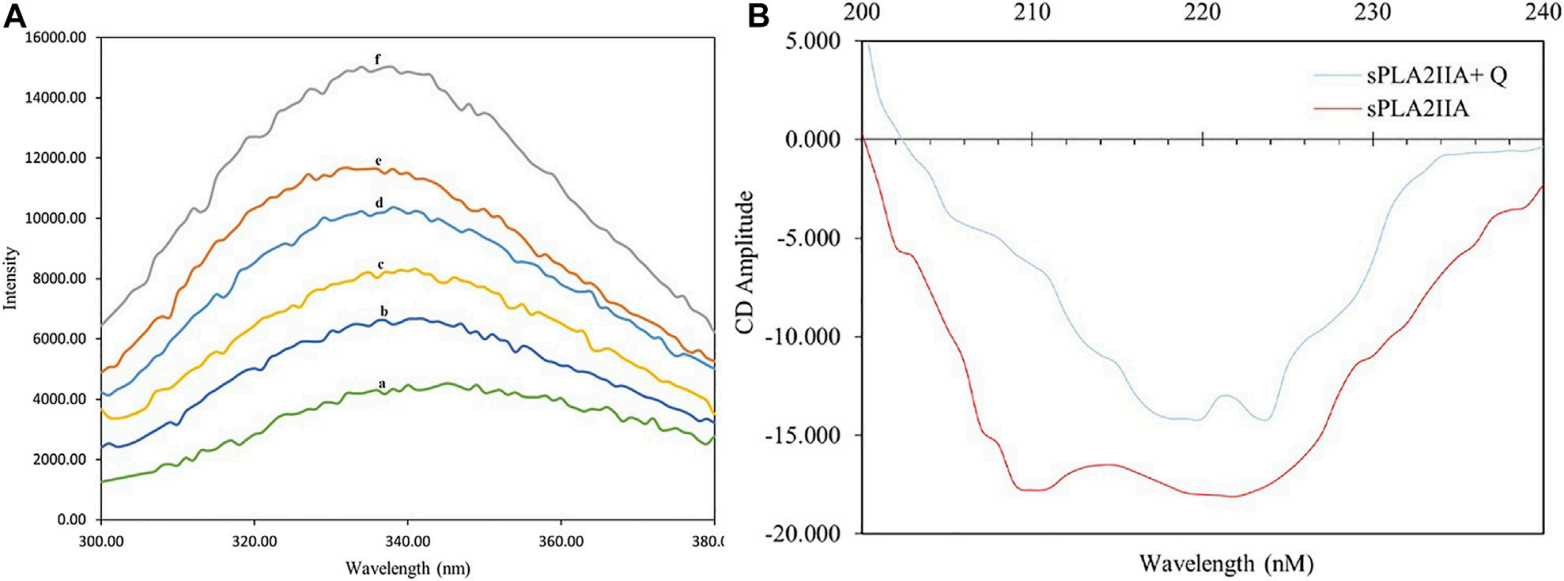
Intrinsic fluorescence and circular dichroism studies. **(A)** Intrinsic fluorescence spectra of sPLA_2_IIa enzyme **(a)**, sPLA_2_IIa enzyme with quercitrin of 0.02 µM **(b)**, 0.04 µM **(c)**, 0.06 µM **(d)**, 0.08 µM **(e)**, and 0.1 µM **(f)** concentrations. **(B)** UV-CD spectra of sPLA_2_IIa enzyme with and without IC_50_ concentration of quercitrin between 200–240 nm.

Generally, the interaction of inhibitors with the sPLA_2_IIa enzyme leads to exposure of amino acids such as phenylalanine, tryptophan, and tyrosine to the surrounding media, altering the intrinsic fluorescence of the enzyme (Toyama, et al., 2014). This alteration indicates structural changes in proteins due to substrate or ligand interaction. The increased fluorescence intensity of sPLA_2_IIa upon quercitrin binding confirmed the formation of enzyme-inhibitor complexes (Figure 4A). Increased relative intrinsic fluorescence was observed by increasing the quercitrin concentration from 0.02 to 0.1 µM. The results indicated that quercitrin directly interacts with sPLA_2_IIa enzyme.

The far UV-CD study reveals the changes in sPLA_2_IIa structure upon binding to an inhibitor (Kelly et al., 2005), which validates the findings of the fluorescence study. The far UV-CD spectrum of sPLA_2_IIa without inhibitors gave two characteristic negative bands at 210 and 222 nm. The spectrum of sPLA_2_IIa in the presence of an IC_50_ concentration of quercitrin in the reaction mixture showed diminished peak height and shifted to the higher wavelengths of 220 and 224 nm, respectively (Figure 4B). The percentage of the secondary structure of sPLA_2_IIa was calculated using K2D3 software. Quercitrin, upon binding to the active site of sPLA_2_IIa, reduced the α-helix from 43.02 % to 21.04%, increased β-turn from 11.07 % to 31.55% and the random coil from 45.91 % to 47.41% (Table 2). This significant conformational change in the secondary structure of sPLA_2_IIa indicated direct interaction with the quercitrin.

**TABLE 2:** Effect of quercitrin on secondary structures of sPLA_2_IIA: the secondary structure contents were calculated using K2D3 software.

Secondary structure	sPLA_2_IIa (%)	sPLA_2_IIa + quercitrin (IC_50_)
α-Helix	43.02%	21.04%
β-Turn	11.07%	31.55%
Random coil	45.91%	47.41%

**FIGURE 5:**
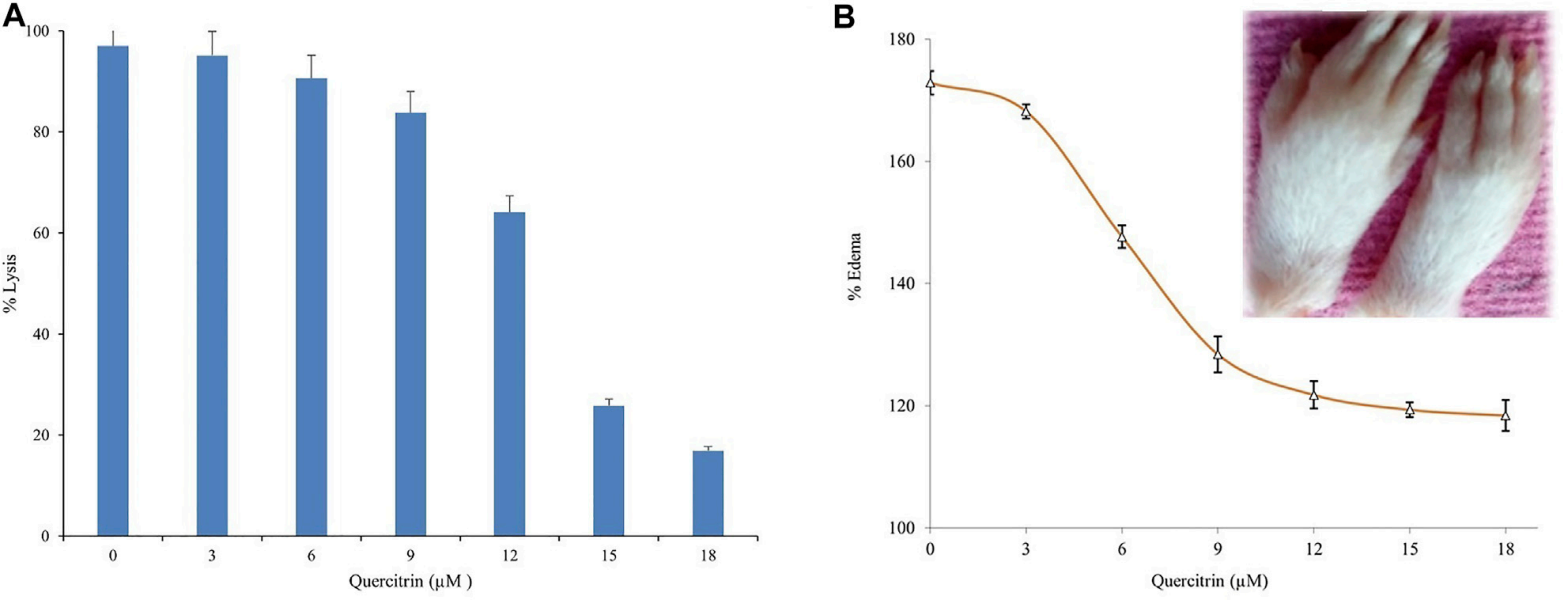
Neutralization of sPLA_2_IIa induced indirect hemolytic activity and edema. **(A)** Quercitrin in the range of 0–18 μM concentration neutralized sPLA_2_IIa induced indirect hemolytic activity in a dose-dependent manner. **(B)** Quercitrin (0–18 µM) neutralized the edema-inducing activity of sPLA_2_IIa in a dose-dependent manner with an apparent shift of IC_50_ value is 8.96 ± 0.16. The saline-injected into the respective left foot pad served as standard control. The data are expressed in mean ± standard deviation (*n* = 3).

We evaluated the efficacy of quercitrin for neutralizing the indirect hemolytic activity of sPLA_2_IIa by treatment in the range of 0 to 18 μg/ml. It was found that quercitrin effectively neutralized sPLA_2_IIa-induced hemolytic activity from 97.32% ± 1.23–16.91% ± 2.03 at 18 μg/ml concentration (Figure 5A). Neutralization sPLA_2_IIa activity by an *in situ* method was according to the *in vitro* inhibitory potential of quercitrin. This demonstrated that sPLA_2_IIa causes cell membrane asymmetry by degrading glycerol phospholipids of the membranes (Granata, et al., 2003).

The *in vitro* studies returned positive results but did not show efficiency by *in vivo* studies due to heterogeneity of the environment. The administration of purified sPLA_2_IIa enzyme from human synovial/pleural fluids into joints induces an inflammatory response with edema, swelling of the synovial cells, and hyperplasia (Fawzy et al., 1988; Weichman et al., 1989; Bomalaski et al., 1991). The inhibitor, quercitrin, was tested for a neutralizing edema-inducing activity of the sPLA_2_IIa enzyme; it reduced inflammatory response (edema) from 172.87% ± 1.9–118.41% ± 2.53 at 18 μM concentration (Figure 5B). The neutralization of edema-inducing activity sPLA_2_IIa by quercitrin was according to the *in vitro* and *in situ* inhibitory potential of quercitrin.

**FIGURE 6:**
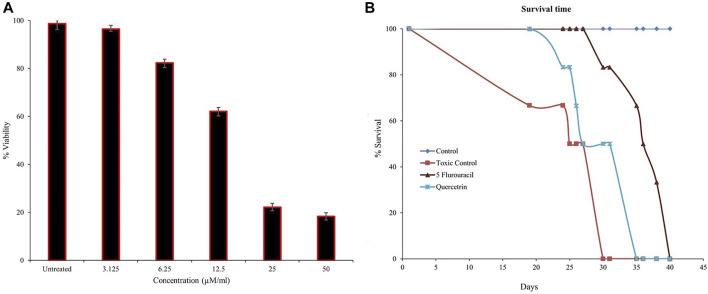
Estimation of cell viability and mean survival time (MST). **(A)** Quercitrin in the range of 0–50 µM concentration reduced PC3 cell viability in a dose-dependent manner and the IC_50_ value was found to be 12.26 μM ± 0.9. **(B)** The graph shows the increased mean survival time of mice bearing Ehrlich ascites carcinoma tumor cells on treatment with quercitrin and standard 5-fluorouracil. The data are expressed in mean ± standard deviation (*n* = 3)

Several sPLA_2_ inhibitors have been explored for cancer prevention and treatment strategies by targeting inflammatory pathways (Lee et al., 2012; Javed et al., 2021; Vecchi et al., 2021). The previous studies showed that quercitrin limits TPA-induced neoplastic transformation in JB6 cells and anticancer effects by inducing apoptosis in the A549 lung cancer cell line (Zheng et al., 2012). Hence, we further experimented to evaluate quercitrin for antiproliferative potency via *in vitro* and *in vivo* models. When we initially tested the effect of quercitrin on PC3 cell viability, quercitrin reduced the PC3 cell viability from 98.66% ± 2.51–18.3% ± 1.52 at 50 µM concentration with the IC_50_ value of 12.26 μM ± 0.9 (Figure 6A).

To better understand the anticancer effect of quercitrin, we established an EAC-induced mouse model. Treatment with quercitrin and 5-fluorouracil (standard) showed protective effects against EAC-induced mortality. Animals treated with quercitrin (G3) showed increased mean survival time (MST) to 35 days compared to disease/toxic control (G2), which showed 30 days, while the standard 5-fluorouracil treatment group (G4) showed a maximum MST of 40 days (Figure 6B). The disease/toxic control group (G2) showed a statistically significant (*p* < 0.05) rise in body weight up to 46.6 ± 4.0 g compared to the normal control (G1) body weight which was 33.3 ± 2.8g, whereas the quercitrin treated group (G3) showed significantly reduced body weight, (*p* < 0.05) at 39.1 ± 3.7g, while the standard treated group (G4) showed 34.8 ± 2.8 g. In addition, G2 showed a viability of EAC cells of 56.4% ± 5.9, whereas the quercitrin treated group (G3) decreased to 48.51% ± 8.7; the standard treated group (G4) showed 50.97% ± 10.48 (Table 3). Along with the survival studies, the ascetic fluid and packed cell volume were used to assist the anticancer efficacy of quercitrin. The ascetic fluid was drawn from the control and the treated animals; the control fluid was 15.3ml, whereas this volume was found to decrease in treated animals to 3.5 ml. This was also supported by the packed cell volume, an indicator of the tumor load, which was also decreased in the treated animals (1.3 ml) compared to the control (9.2 ml) animals. Quercitrin thus displayed significant anticancer activity (*p* < 0.05) against EAC-induced tumors in mice.

**TABLE 3:** Antitumor activity of quercitrin.

Parameters	Group 1: normal control	Group 2: toxic/disease control	Group 3: quercitrin	Group 4: standard (5-flurouracil)
% Viability	NA	56.4 ± 05.9	48.51 ± 8.7	50.9 ± 10.48**
% Survival fraction	33.3 ± 2.8	46.6 ± 4.0	39.1 ± 3.7*	34.8 ± 2.8***
MST	NA	30	35	40

*p < 0.05, **p < 0.01, ***p < 0.01 compared to the EAC control.

**FIGURE 7:**
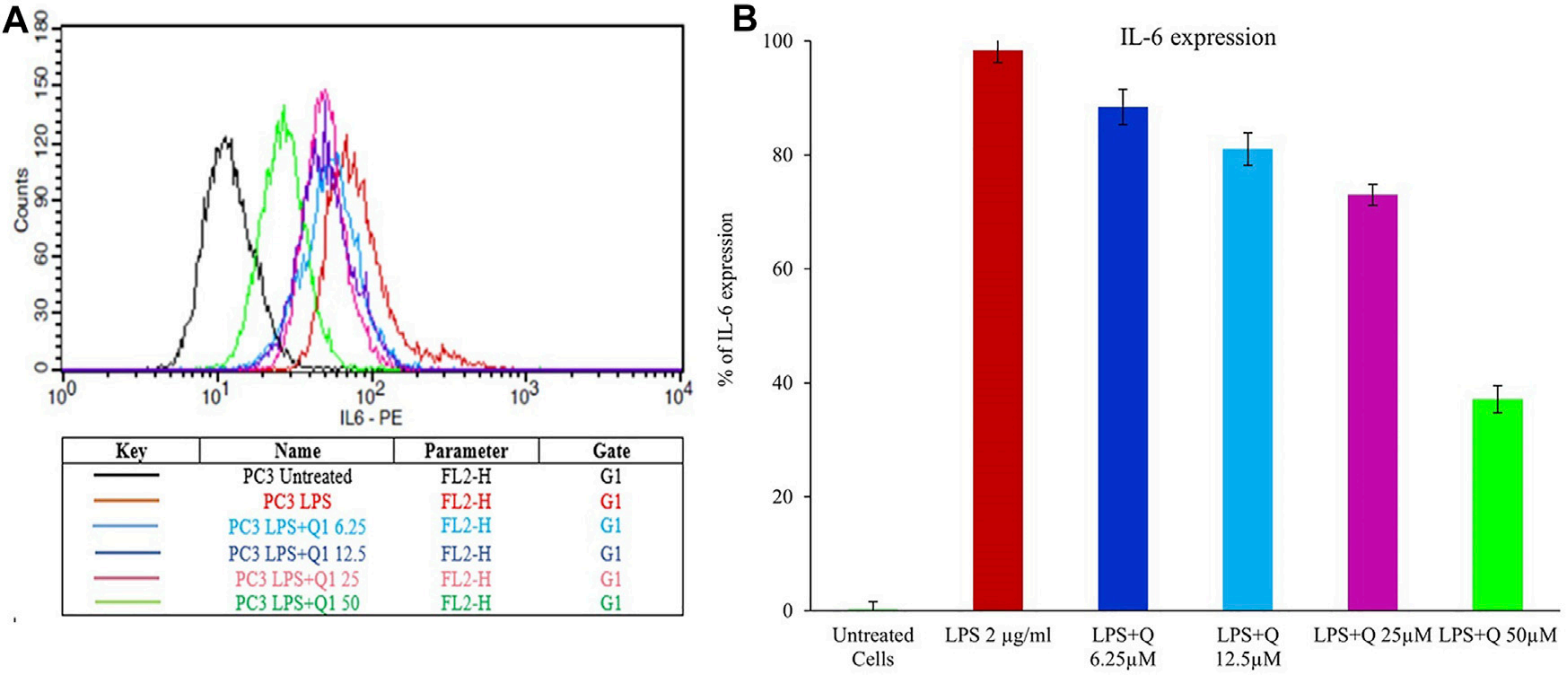
Estimation of the effect of quercitrin on IL6 expression. **(A)** Graph of mean fluorescence intensities of IL-6 stained with anti IL6 antibody, which is conjugated with phycoerythrin fluorochrome upon treatment of quercitrin (0–50 µM) in LPS treated PC3 cell line. **(B)** The bar graph represents the % of IL-6 expression upon treatment of quercitrin.

The elevated concentrations of IL-6 in the tumor microenvironment regulate the several signaling pathways of cancer, including apoptosis, proliferation, angiogenesis, invasiveness, and metastasis. IL-6 is known to activate numerous signaling cascades such as the JAK-STAT pathway, the p38 MAPK pathway, the ERK pathway, and the PI3-kinase pathway, reflecting the strong correlation between inflammation and cancer (Jones, Scheller, and Rose-John, 2011). The elevated level of IL-6 correlates with prostate cancer burden (Hirano, 1992; Vainer et al., 2018). Therefore, the effect of quercitrin on the expression of IL-6 in LPS treated PC3 cell lines was tested. Treating LPS (2 μg/ml) to the PC3 cell line resulted in an increase in the percentage of cells expressing IL-6—98.35% ± 2.2. Treatment with different concentrations of quercitrin (6.25, 12.5, 25, and 50 µM) reduced the IL-6 concentration from 98.35% ± 2.2–37.12% ± 2.4 (Figure 7). Hence, it was anticipated that targeting IL-6 would constitute a novel treatment strategy for various immune-mediated diseases and cancers.

## Conclusion

An effective anti-inflammatory drug with anticancer activity is needed to treat cancers. Quercitrin inhibited inflammatory sPLA_2_IIa enzyme *in vitro* and neutralized sPLA_2_IIa-induced mouse paw edema, thus proving itself to be an effective anti-inflammatory molecule. Quercitrin significantly reduced the cell viability and inflammatory cytokine IL-6 in the PC3 cell line and increased the mean survival time in the EAC-bearing mice model. It displayed drug-like qualities by ADME toxicity analysis, which is mandatory for ensuring its use as a drug. Thus, quercitrin has shown promising anti-inflammatory and anticancer effects.

## Data Availability

The raw data supporting the conclusion of this article will be made available by the authors, without undue reservation.
